# Sulforaphane Induces Oxidative Stress and Death by p53-Independent Mechanism: Implication of Impaired Glutathione Recycling

**DOI:** 10.1371/journal.pone.0092980

**Published:** 2014-03-25

**Authors:** José Miguel P. Ferreira de Oliveira, Maria Costa, Tiago Pedrosa, Pedro Pinto, Catarina Remédios, Helena Oliveira, Francisco Pimentel, Luís Almeida, Conceição Santos

**Affiliations:** 1 CESAM & Laboratory of Biotechnology and Cytomics, Department of Biology, University of Aveiro, Campus Universitário de Santiago, Aveiro, Portugal; 2 Lenitudes, Lisboa, Portugal; 3 Faculty of Health Sciences, University Fernando Pessoa, R. Carlos da Maia 296, Porto, Portugal; 4 Center for Health Studies & Research, University of Coimbra, Avenida Dias da Silva, Coimbra, Portugal; 5 Faculdade de Ciências da Saúde - Universidade da Beira Interior, Avenida Infante D. Henrique, Covilhã, Portugal; 6 Luzitin SA, R. Bayer 16, Coimbra, Portugal; 7 Blueclinical Phase I, R. Sarmento de Beires 153, Porto, Portugal; The University of Tokyo, Japan

## Abstract

Sulforaphane (SFN) is a naturally-occurring isothiocyanate best known for its role as an indirect antioxidant. Notwithstanding, in different cancer cell lines, SFN may promote the accumulation of reactive oxygen species (ROS) and cause cell death e.g. by apoptosis. Osteosarcoma often becomes chemoresistant, and new molecular targets to prevent drug resistance are needed. Here, we aimed to determine the effect of SFN on ROS levels and to identify key biomarkers leading to ROS unbalance and apoptosis in the p53-null MG-63 osteosarcoma cell line. MG-63 cells were exposed to SFN for up to 48 h. At 10 μM concentration or higher, SFN decreased cell viability, increased the%early apoptotic cells and increased caspase 3 activity. At these higher doses, SFN increased ROS levels, which correlated with apoptotic endpoints and cell viability decline. In exposed cells, gene expression analysis revealed only partial induction of phase-2 detoxification genes. More importantly, SFN inhibited ROS-scavenging enzymes and impaired glutathione recycling, as evidenced by inhibition of glutathione reductase (GR) activity and combined inhibition of glutathione peroxidase (GPx) gene expression and enzyme activity. In conclusion, SFN induced oxidative stress and apoptosis via a p53-independent mechanism. GPx expression and activity were found associated with ROS accumulation in MG-63 cells and are potential biomarkers for the efficacy of ROS-inducing agents e.g. as co-adjuvant drugs in osteosarcoma.

## Introduction

Osteosarcoma is the most frequent primary solid malignancy of the bone and shows higher incidence in children, adolescents and young adults [1], [2]. The overall survival of nonmetastatic osteosarcoma patients has improved substantially with the introduction of adjuvant and neoadjuvant chemotherapy regimens. However, to improve the prognosis of patients with detectable metastatic, recurrent or nonresectable osteosarcoma, more selective and potent drugs need to be developed [3], [4], [5], [6].

Epidemiological data continue to show that dietary intake of cruciferous vegetables (Brassicaceae) may protect against carcinogenesis, reviewed in [7], [8]. Sulforaphane (SFN), a natural isothiocyanate found in Brassicaceae, has been shown to possess anticancer and anti-inflammatory activities in many cancer cell lines [9], [10], [11], [12]. SFN is best known for its role as an indirect antioxidant, as it induces several phase 2 detoxification enzymes [13], [14] and inhibits procarcinogenic phase 1 enzymes [15]. This isothiocyanate can decrease cell proliferation by causing cell cycle arrest and inducing apoptosis [12], [16], [17]. In tumour cells, SFN may induce apoptosis by death receptor 5, activator protein 1, mitogen-activated protein kinases or mitochondrial dysfunction, and additionally SFN may suppress concurring prosurvival pathways, e.g. via active inhibition of the nuclear factor-kappa B activation [17], [18], [19]. Other potential mechanism of SFN action via SFN-conjugates is histone deacetylase inhibition, which was shown to increase histone acetylation at the promoters of p21 and Bax, and was associated with cell cycle arrest and apoptosis [20], [21]. In osteosarcoma, SFN has been found to induce apoptosis via activation of the death-receptor pathway [17]. Despite its role as an indirect antioxidant and inducer of Antioxidant Response Element (ARE) genes, there is evidence that exposure to SFN results in a transient reactive oxygen species (ROS) burst, of which the duration and magnitude are both dependent on the SFN concentration and exposure period. In different cancer cell lines it has been reported that activation of apoptosis by SFN is highly dependent on ROS generation, as the apoptotic effect could be counteracted with ectopic catalase (Cat) expression [22], [23], [24], [25], [26]. Recent studies have shown that cells with low mitochondrial respiratory chain activity are mostly protected from SFN-induced DNA breakage, G2/M phase arrest, disruption of mitochondrial membrane potential and apoptosis [23], [26], [27]. These observations reinforced the notion that the mitochondrial respiratory chain is the main site for SFN-induced ROS production and subsequent ROS-induced cellular alterations. Overall, the development of drugs targeting ROS-sensitive cancer cells shows much potential to chemotherapy [28], [29].

In osteosarcoma, wild-type p53 function is frequently altered or entirely absent [2]. Several anticancer agents, e.g. etoposide or 5-fluorouracil, however, predominantly induce apoptosis via a p53-dependent mechanism [30] and this action may render these agents less effective in p53-deficient osteosarcoma therapy.

The aims of this work are to test SFN efficacy in inducing ROS in a p53-null osteosarcoma cell line, and to evaluate the most sensitive biomarkers to assess oxidative stress within this model. For this, the p53-null model cell line MG-63was exposed to SFN and several parameters related to oxidative state were assessed and correlated with cytotoxicity and apoptosis induced by SFN treatment.

## Materials and Methods

### Cell Culture and Exposure Treatment

All cell culture reagents were purchased from Life Technologies (Carlsbad, CA-USA), unless otherwise stated. Human osteosarcoma MG-63 cell line (ATCC, Manassas, VA-USA) was cultured in α-Minimum Essential Medium supplemented with 10% foetal bovine serum, 2.5 μg/ml fungizone, and 100 U/ml penicillin-100 μg/ml streptomycin at 37°C in a humidified atmosphere containing 5% CO_2_. When ∼80% cell confluence was reached, cells were trypsinised with Trypsin-EDTA (0.25% Trypsin, 1 mM EDTA) and subcultured at a split ratio of 1∶10. D,L-sulforaphane (SFN; Sigma-Aldrich, St. Louis, MO-USA) was dissolved in DMSO (Sigma-Aldrich, St. Louis, MO-USA) at a 10 mM stock concentration and stored at −20°C. Cells were allowed to adhere for 24 h and medium was replaced with fresh medium containing 0, 5, 10, and 20 μM SFN. Cells were exposed for 24 and 48 h.

### Cell Morphology and Confluence

Throughout the experiment, cultures were routinely visualised for confluence and cell morphology. Control and SFN-exposed MG-63 cells were daily observed under inverted microscopy in a Nikon Eclipse TS100 microscope (Nikon, Tokyo, Japan) for confluence and changes in morphology between control and exposed cells.

### Cell Viability and Apoptosis

Cell viability and apoptosis were analysed by flow cytometry (FCM) in a Coulter Epics XL Flow Cytometer (Beckman Coulter, Hialeah, FL-USA), using the FITC Annexin V Apoptosis Detection Kit I (BD Pharmingen, San Diego, CA-USA) as recommended by the manufacturer. Briefly, cells were harvested and washed with PBS, pH 7.2. Cells were resuspended in diluted binding buffer provided with the kit (1∶10 in distilled water) at 1×10^6^ cells/ml. Five microliters FITC-Annexin V and 5 μl propidium iodide (PI; Sigma-Aldrich; St. Luis, MO-USA) were used to stain 100 μl cell suspension for 15 min at room temperature in the dark, after which each sample was diluted in 400 μl binding buffer. At least 10,000 events were analysed for each sample and percentages were calculated from the number of cells in each quadrant divided by the total number of cells.

### Determination of Caspase-3 Activity

Caspase-3 activity was determined using the APOPCYTO Caspase-3 Colorimetric Assay Kit (MBL, Nagoya, Japan), with few modifications. In brief, control cells or cells exposed to 10 or 20 μM SFN for 48 h were washed with PBS and collected after trypsinization. Cells (1.5×10^6^) were collected by centrifugation, resuspended in 100 μl cell lysis buffer provided with the kit and incubated on ice for 10 min. After centrifugation at 10,000 g, 4°C for 5 min, the cleared cell extract was collected and placed on ice and total protein concentration was determined (see below, under Protein quantification subsection) The final reaction contained 1 vol. 2× reaction buffer containing 10 mM DTT : 1 vol. cell extract : 0.1 vol. 10 mM Caspase-3 substrate DEVD-*p*-nitroanilide. Inhibition control reactions containing 10 μM DEVD-FMK inhibitor were also performed. Microplates were incubated at 37°C, in the dark for 18 h, and A405 nm was subsequently measured in 96-well plates in a Synergy HT Multi-mode Microplate Reader (BioTek Instruments, Winooski, VT-USA). Caspase-3 activity was extrapolated from a *p*-nitroanilide substrate standard curve, and caspase-3 specific activity was calculated according to the manufacturer’s protocol.

### Total Antioxidant Activity (TAA)

For the TAA assay, the Antioxidant Assay Kit (Sigma-Aldrich, St. Louis, MO-USA) was used. Cell homogenates were prepared as described by Quick and co-workers [31] with modifications. Briefly, cells were scraped in cold PBS and centrifuged at 1,000 g, for 10 min, at 4°C. The cell pellet was resuspended in assay buffer and sonicated for 30 s. The homogenates were centrifuged at 12,000 g for 15 min at 4°C, and the supernatants were stored at −80°C until further analysis. Reaction with 2,2′-azino-bis(3-ethylbenzthiazoline-6-sulfonic acid) (ABTS) and spectrophotometric measurements were according to the kit manufacturer’s instructions.

### Determination of Intracellular Reduced Glutathione (GSH) Levels

For GSH quantification, the Glutathione Assay Kit, Fluorimetric (Sigma-Aldrich, St. Louis, MO-USA) was used. In brief, 10^5^ cells/ml were seeded in a fluorimetric 96-well plate. After exposure, cells were washed with PBS, the kit reagents were added and fluorescence was measured on microplate reader at 360-nm excitation and 485-nm emission. In parallel with sample measurement, a calibration curve was performed with GSH standard to extrapolate sample concentration. In order to normalise GSH levels, total protein content was determined for each sample. After fluorimetric reading, cells were washed with PBS, incubated with the Glutathione Assay Kit’s lysis buffer during 30 min in shaker. After this, 5 μl of homogenate were taken to a new well and total protein content was determined for each sample (see below, under Protein quantification subsection).

### Intracellular ROS Formation

Intracellular ROS production was assessed by FCM with the use of dichlorodihydrofluorescein diacetate (Sigma-Aldrich, St. Louis, MO-USA) fluorescent probe. After SFN exposure, medium was discarded and cells were incubated for 30 min, at 37°C, in the dark with serum-free α-MEM containing 10 μM dichlorodihydrofluorescein diacetate. Cells were washed with PBS, trypsinised, and collected for analysis. ROS formation was estimated from the median fluorescence intensity (MFI) of dichlorofluorescein using the FlowJo software (Tree Star Inc., Ashland, OR-USA).

### Enzyme Activity Assays

Adherent subconfluent cells were washed with PBS and scraped. Cells were collected by centrifugation, resuspended in a variable volume of cold 5 mM phosphate buffer (5 mM potassium phosphate, 1 mM EDTA, pH 7.4) and sonicated for 30 s. After centrifugation at 12,000 g, 4°C for 15 min, cleared cell extracts were collected.

For Cat, superoxide dismutase (SOD) and glutathione reductase (GR) enzyme assays, ∼ 5×10^6^ cells were resuspended in 750 μl cold 5 mM phosphate buffer and assayed at 25°C. From the cleared cell extracts, Cat activity was determined by monitoring oxygen formation from H_2_O_2_ decay. For this, 25 μl of each cleared cell extract were added to 925 μl phosphate buffer (50 mM potassium phosphate, 5 mM EDTA, pH 7.4). Background O_2_ formation was determined in an Oxygraph System instrument (Hansatech Instruments, Norfolk, UK), and subsequently 50 μl H_2_O_2_ were added. Cat activity was determined from H_2_O_2_ conversion to O_2_, normalised to background O_2_ formation. Total SOD activity was determined using the SOD-assay kit (Sigma-Aldrich, St. Louis, MO-USA) and A440 nm was followed. GR activity assay was carried out according to Dringen and Gutterer [32] with some modifications. The reaction was carried in 100 mM phosphate buffer (100 mM potassium phosphate, 1 mM EDTA, pH 7.4) and additionally contained 0.2 mM NADPH (Sigma-Aldrich, St. Louis, MO-USA) and 1 mM glutathione disulfide (GSSG; Sigma-Aldrich, St. Louis, MO-USA), in a final 320-μl volume. A GR calibration curve was used to determine GR activity in samples.

For the GPx enzyme assay, ∼5×10^6^ cells were resuspended in 350 μl cold 5 mM phosphate buffer and sonicated for 30 s once on ice. From the cleared cell extracts, GPx activity was determined as described by Smith and Levander [33] with some alterations. Briefly, cleared cell extract (50 μl) prepared as described above was diluted in phosphate buffer to the final concentrations 50 mM potassium phosphate, 5 mM of EDTA, pH 7.4, containing 2 mM GSH, 1 mM sodium azide (Sigma-Aldrich, St. Louis, MO-USA), 0.4 mM NADPH and 2 U/ml GR (Sigma-Aldrich, St. Louis, MO-USA). The reaction was initiated by the addition of 10 μl *tert*-butyl hydroperoxide solution (Sigma-Aldrich, St. Louis, MO-USA), incubated at room temperature and A340 nm was measured. SOD, GR and GPx enzyme assays were carried out in 96-well plates in a microplate reader.

### Protein Quantification

Total protein quantification was done using Bradford Reagent (Sigma-Aldrich, St. Louis, MO-USA) and following the manufacturer’s instructions. Five microliters of sample were taken to a 96-well plate and 250 μl of Bradford Reagent were added. The plate remained in agitation in darkness for 10 min and protein content was determined after this period.

### Assessment of mRNA Expression

Gene-specific primers (Table 1) were designed using the Primer3 design tool [34] and were tested for unique hits in the human genome by the UCSC In-Silico PCR tool (http://genome.ucsc.edu/cgi-bin/hgPcr?command=start). RNA was extracted from MG-63 control cells and cells exposed to 10 μM SFN for 48 h, using the TRIzol method. Organic phase separation was achieved in Phase Lock Gel Heavy tubes (5 PRIME Inc., Boulder, CO-USA). The aqueous phase was mixed with 1 vol. 70% ethanol and RNA was purified using RNeasy Mini Kit columns (Qiagen, Hilden, Germany). For cDNA synthesis, 2 μg total RNA were pre-incubated with DNase I (Sigma-Aldrich, St. Louis, MO-USA), DNase I was inactivated and total RNA was reverse-transcribed with 1 mM Oligo (dT)_18_, using the Omniscript RT Kit (Qiagen, Hilden, Germany). The cDNA samples were prediluted in ultrapure MilliQ water (1∶20). The final individual qPCR reactions contained iQ SYBR Green Supermix (BioRad, Hercules, CA-USA), 1.5 μM each gene-specific primer and 1∶4 (v/v) prediluted cDNA (1∶20). The qPCR program included 1 min denaturation at 95°C, followed by 40 cycles at 94°C for 5 s, 58°C for 15 s, and 72°C for 15 s. After qPCR, a melting temperature program was performed. At least three qPCR technical replicates were performed per sample from each of two independent biological assays. Average PCR efficiencies and cycle thresholds were estimated from the fluorescence data using the algorithm Real-Time PCR Miner [35]. The estimated average efficiencies and cycle thresholds were used to determine gene expression of exposed cells relative to control cells and normalised with the GAPDH reference gene, following the Pfaffl method [36].

**Table 1:** Oligonucleotide primers used for qPCR.

Target gene	Forward primer (5′-3′)	Reverse primer (5′-3′)
*CAT*	TGAACTGTCCCTACCGTGCT	TATTGGATGCTGTGCTCCAG
*GAPDH*	ACACCCACTCCTCCACCTTT	TACTCCTTGGAGGCCATGTG
*GPX1*	CGGGACTACACCCAGATGAA	TCTCTTCGTTCTTGGCGTTC
*GSTM1*	CAGAGCAACGCCATCTTGT	GCCAGCTGCATATGGTTGT
*GSTM4*	AGAGCAACGCCATCCTGT	GATTGGAGACGTCCATAGCC
*GSR*	GATCCCAAGCCCACAATAGA	TCGCTGGTTATTCCTAAGCTG
*NQO1*	GCACTGATCGTACTGGCTCAC	GACTCCACCACCTCCCATC
*SOD1*	GGTGTGGCCGATGTGTCTAT	TTCCAGCGTTTCCTGTCTTT
*SOD2*	CCCTGGAACCTCACATCAAC	CTGAAGAGCTATCTGGGCTGTAA
*TXNRD1*	GTGGGCTTTCACGTACTGG	CTGCACAGACAGGGTGGA

### Correlation Analysis

Pearson correlation analysis was performed to investigate the association between different parameters related to oxidative stress and apoptosis in cells exposed to 0, 5, 10, and 20 μM SFN for 24 and 48 h. Correlations were considered significant for p<0.05.

### Statistical Analysis

For most quantitative assays, three independent assays with at least three technical replicates were performed. For qPCR analysis and caspase-3 activity assay, two independent assays with at least three replicates were performed. For determination of intracellular GSH levels, two independent assays with two replicates each were considered. The statistical analysis was performed using SigmaPlot for Windows version 11.0 (Systat Software Inc., San Jose, CA-USA). Statistical significance between control and SFN-treated groups was evaluated by one-way or two-way ANOVA followed by Holm-Sidak’s test. When necessary, data were transformed to achieve normality and equality of variances. The differences were considered significant for *p*<*0.*05. The data were expressed as mean ± SEM. Pearson’s correlations for the tested endpoints were considered significant for *p*<0.05 and *p*<0.01.

## Results

### SFN Induces Morphological Changes, Apoptosis and Viability Loss in MG-63 Cells

**Figure 1:**
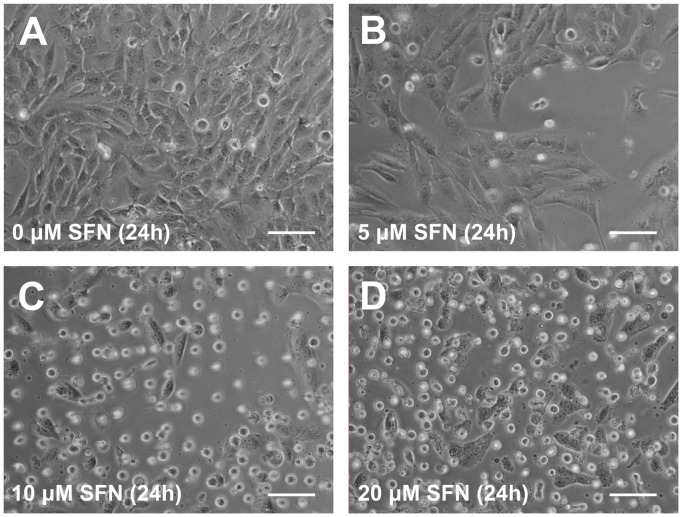
Effect of SFN treatment on cell morphology. Cells were exposed for 24(A) Control cells. (B–D) Cells exposed to 5, 10, or 20 μM SFN respectively. Scale bar: 100 μm.

Relative to control, cells treated with SFN showed morphological alterations, such as cell enlargement and loss of adherence, which were more noticeable for exposure to 10 and 20 μM SFN, as visualised by inverted microscopy (Fig. 1). Moreover, the presence of SFN resulted in a concentration-dependent decrease in the number of cells with exposure time.

**Figure 2:**
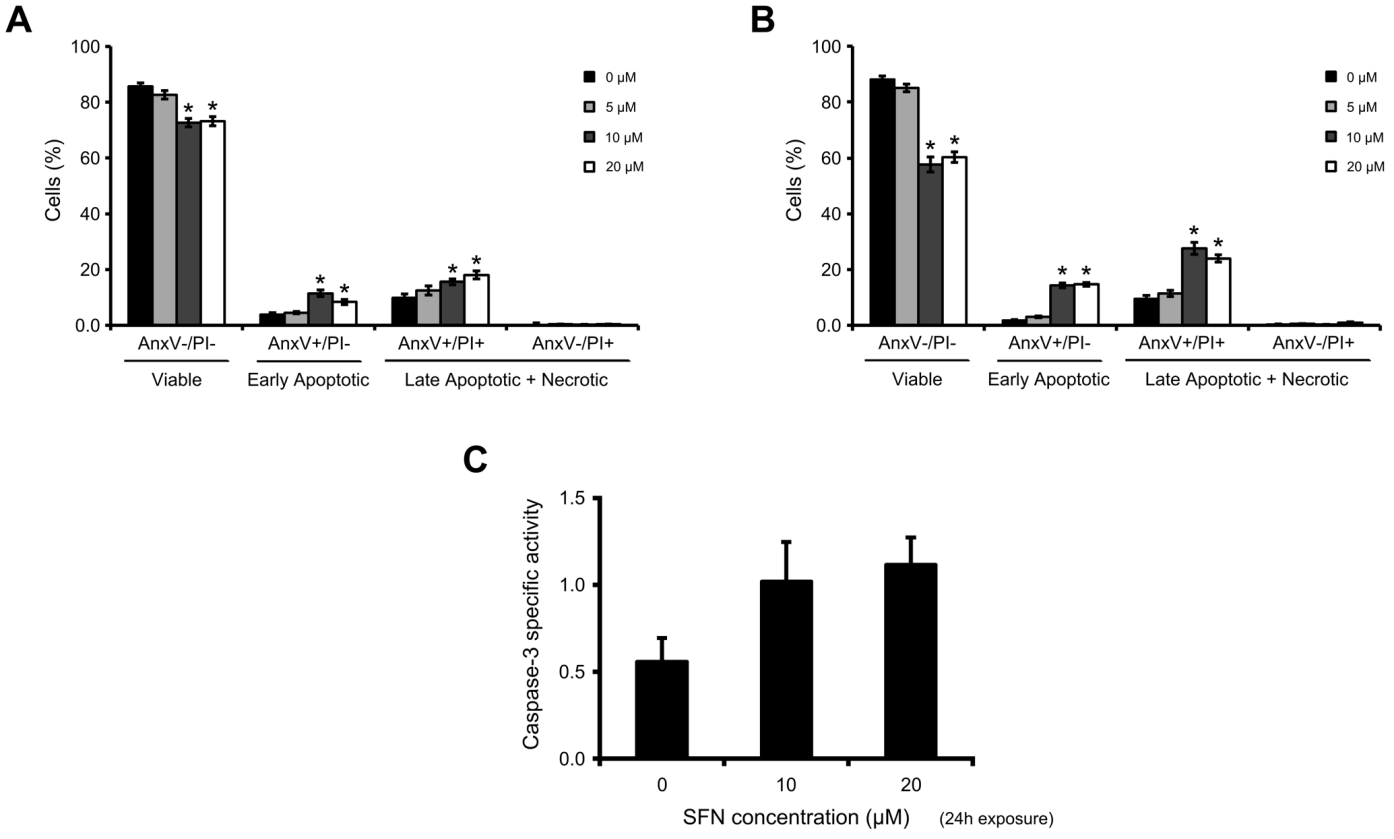
Cytotoxicity and apoptosis induction by SFN. (A, B) Cells were incubated with FITC-Annexin V conjugate and PI after SFN exposure for 24 or 48 h respectively. Data shown are mean ± SEM (n = 3). *, significantly different between control and SFN-treated cells (*p*<0.05). (C) Caspase-3 activity. Cells were exposed for 48 h, the cleared cell extracts were incubated with DEVD-*p-*nitroanilide and caspase-3 activity was measured spectrophotometrically. Data shown are mean ± SEM (n = 2).

In addition to the observed altered morphology and lower cell number, SFN decreased the%viable cells in a concentration-dependent manner, as determined by incubation with FITC-Annexin V conjugate and PI (Fig. 2A, B). Noteworthy, a sharp decline was observed in cell viability from 5 to 10 μM SFN exposure, particularly for the 48-h exposure period. For the 10 and 20 μM concentrations, SFN exposure increased the%cells in early or late apoptosis/necrosis. For these concentrations, cells additionally showed an increase in caspase-3 activity (Fig. 2C).

### SFN Induces a General Antioxidant Response but Decreases Intracellular GSH Levels in MG-63 Cells

**Figure 3:**
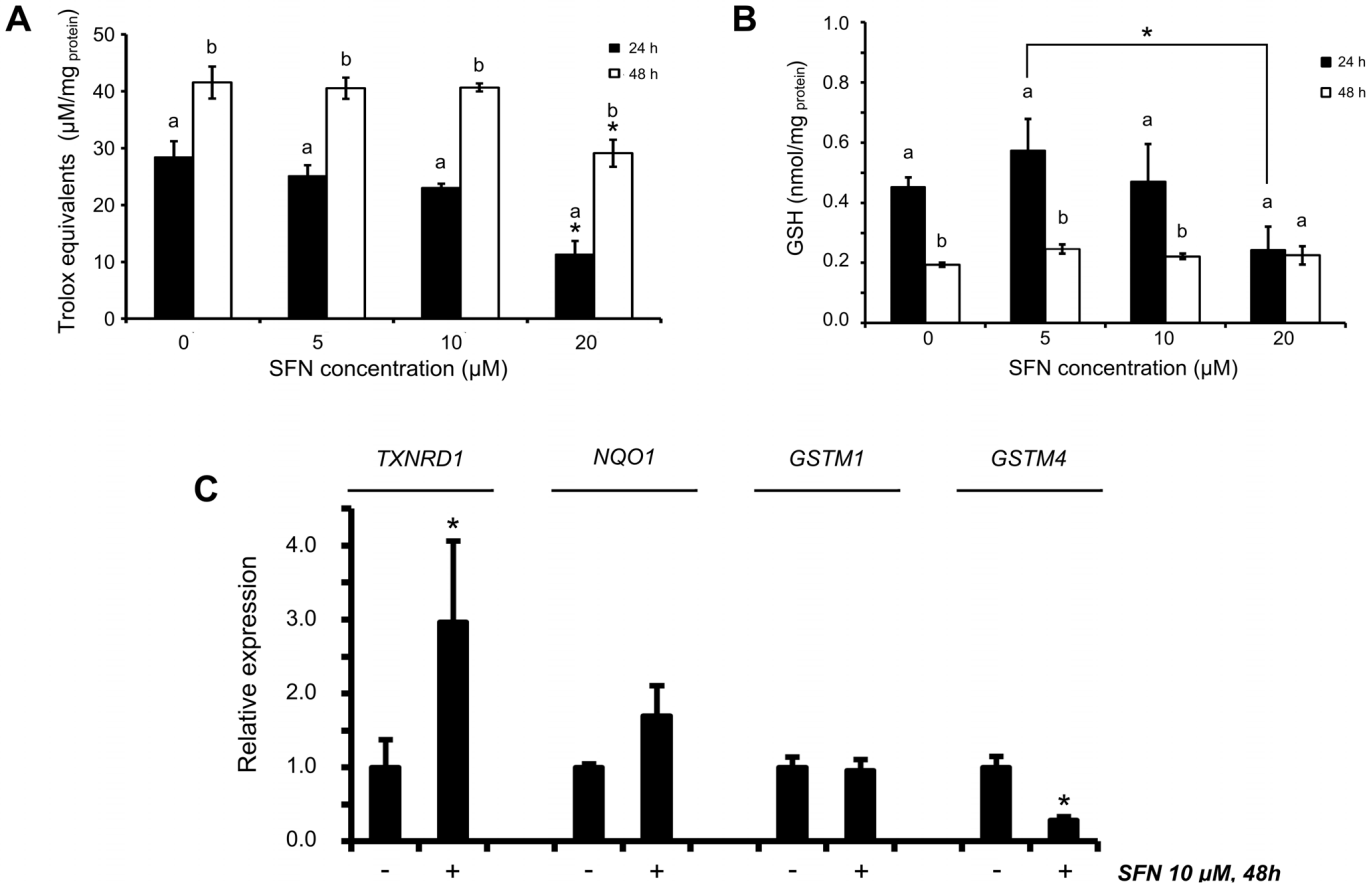
Antioxidant state after SFN treatment. (A) Total antioxidant activity (TAA). TAA was determined spectrophotometrically from cell extracts incubated with ABTS reagent. Data shown are mean ± SEM (n = 3). *, significantly different between control and SFN-treated cells (*p*<0.05). a,b, significantly different between times (*p*<0.05). (B) Intracellular GSH levels for the SFN concentrations and exposure times indicated. Data shown are mean ± SEM (n = 2). *, significantly different between the indicated groups (*p*<0.05). a,b, significantly different between times (*p*<0.05). (C) Relative gene expression of selected phase 2 enzymes exposed to 10 uM SFN for 48 h. Data shown are mean ± SEM (n = 2). *, significantly different between control and SFN-treated cells (*p*<0.05).

The intracellular redox balance is mostly determined by the action of specific oxidoreductases and antioxidants, e.g. the reduced non-protein thiol GSH. In this study, exposure to 20 μM SFN resulted in a significant decrease in the intracellular antioxidant activity (Fig. 3A) and was associated with decreased intracellular GSH levels for the 24-h exposure period (Fig. 3B). Although the antioxidant activity increased from 24 to 48 h exposure, intracellular GSH decreased within the same period. This effect was independent of SFN exposure, as also the control cells exhibited this trend, suggesting that intracellular GSH levels decreased over time and other antioxidants e. g. thioredoxin took over the role of GSH.

The antioxidant response was also analysed at the transcriptional level. SFN is a well-known inducer of phase 2 enzymes via the Nrf2 transcriptional activator. In this study, the *TXNRD1* gene, encoding thioredoxin reductase 1 which functions in the general antioxidant response, was induced by SFN treatment (Fig. 3C). Moreover, a slight increase was observed in the expression of *NQO1*, encoding NAD(P)H:quinone oxidoreductase 1, a flavoprotein that catalyses a 2-electron reduction of quinone in the electron respiratory chain and functions as a superoxide scavenger. In contrast to this, the expression of genes encoding the two ubiquitous glutathione-S-transferases M1 and M4 was not increased by SFN treatment and *GSTM4* expression was found decreased after SFN treatment. Collectively, these results indicated that although SFN induce an antioxidant response, the increasing concentrations up to 20 μM SFN resulted in gradually decreased antioxidant defenses in MG-63 cells, associated with depletion of intracellular GSH.

### SFN Treatment Increases Intracellular ROS Formation and Inhibits ROS-scavenging Enzymes

**Figure 4:**
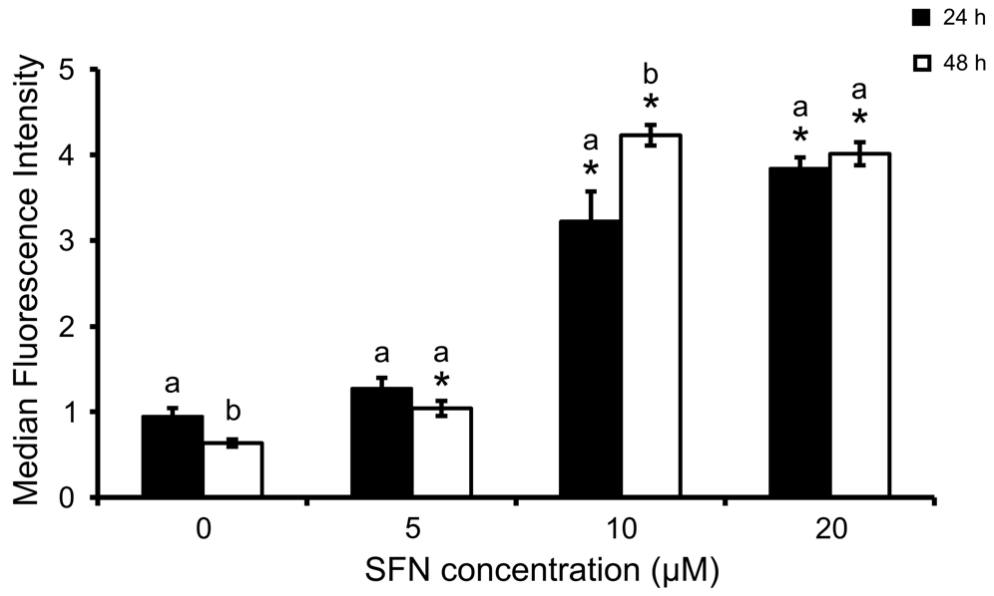
ROS accumulation after SFN treatment. Cells were incubated with10 μM dichlorodihydrofluorescein diacetate and ROS accumulation was estimated from MFI by FCM. Data shown are mean ± SEM (n = 3). *, significantly different between control and SFN-treated cells (*p*<0.05). a,b, significantly different between times (*p*<0.05).

SFN treatment induced ROS accumulation in a concentration-dependent manner (Fig. 4), an observation that was confirmed by correlation analysis (*p*<0.01; Table 2). The increase in intracellular ROS was not gradual between the 5 and 10 μM SFN exposure, and a sharp increase was observed particularly for the 48-h exposure period. Moreover, for 10 μM SFN, longer exposure times resulted in larger accumulation of intracellular ROS.

**Table 2:** Correlation analysis of SFN treatment, oxidative stress and apoptosis.

	|SFN|	Time	ROS levels	SOD activity	Cat activity	GPx activity	GR activity	GSH levels	TAA	Caspase 3 activity	% EA	% LA+Nec	% Viable cells
**|SFN|**	–	n.a.	0.870**	−0.883**	−0.474	−0.876**	−0.756*	**−**0.321	**−**0.554	0.936	0.744	0.719*	**−**0.261
**Time**		–	0.054	**−**0.344	−0.865**	0.198	0.388	−0.779*	0.808*	n.a.	0.146	0.334	**−**0.746
**ROS levels**			–	−0.801*	**−**0.532	−0.885**	−0.807*	**−**0.297	**−**0.360	0.999*	0.941**	0.915**	−0.942**
**SOD activity**				–	0.720*	0.672	0.604	0.603	0.229	**−**0.739	−0.721*	−0.795*	0.782*
**Cat activity**					–	0.296	0.088	0.808*	**−**0.465	**−**0.981	**−**0.577	−0.716*	0.672
**GPx activity**						–	0.905**	**−**0.027	0.563	**−**0.991	−0.825*	−0.775*	0.811*
**GR activity**							–	**−**0.215	0.659	**−**0.970	−0.791*	**−**0.687	0.747*
**GSH levels**								–	**−**0.393	**−**0.578	**−**0.195	**−**0.393	0.315
**TAA**									–	**−**0.841	**−**0.182	**−**0.027	0.095
**Caspase 3 activity**										–	0.837	0.991	**−**0.980
**% EA**											–	n.a.	n.a.
**% LA+Nec**												–	n.a.
**% Viable cells**													–

Significant correlations: *p<0.05; **p<0.01; EA: early apoptotic cells; LA: late apoptotic cells; Nec: Necrotic cells; n.a.: not applicable.

Values are Pearson’s correlation coefficients.

The apoptosis-related parameters investigated, *viz.* caspase-3 activity and%early and late apoptosis, were found positively correlated with ROS levels (*p*<0.05; Table 2).

**Figure 5:**
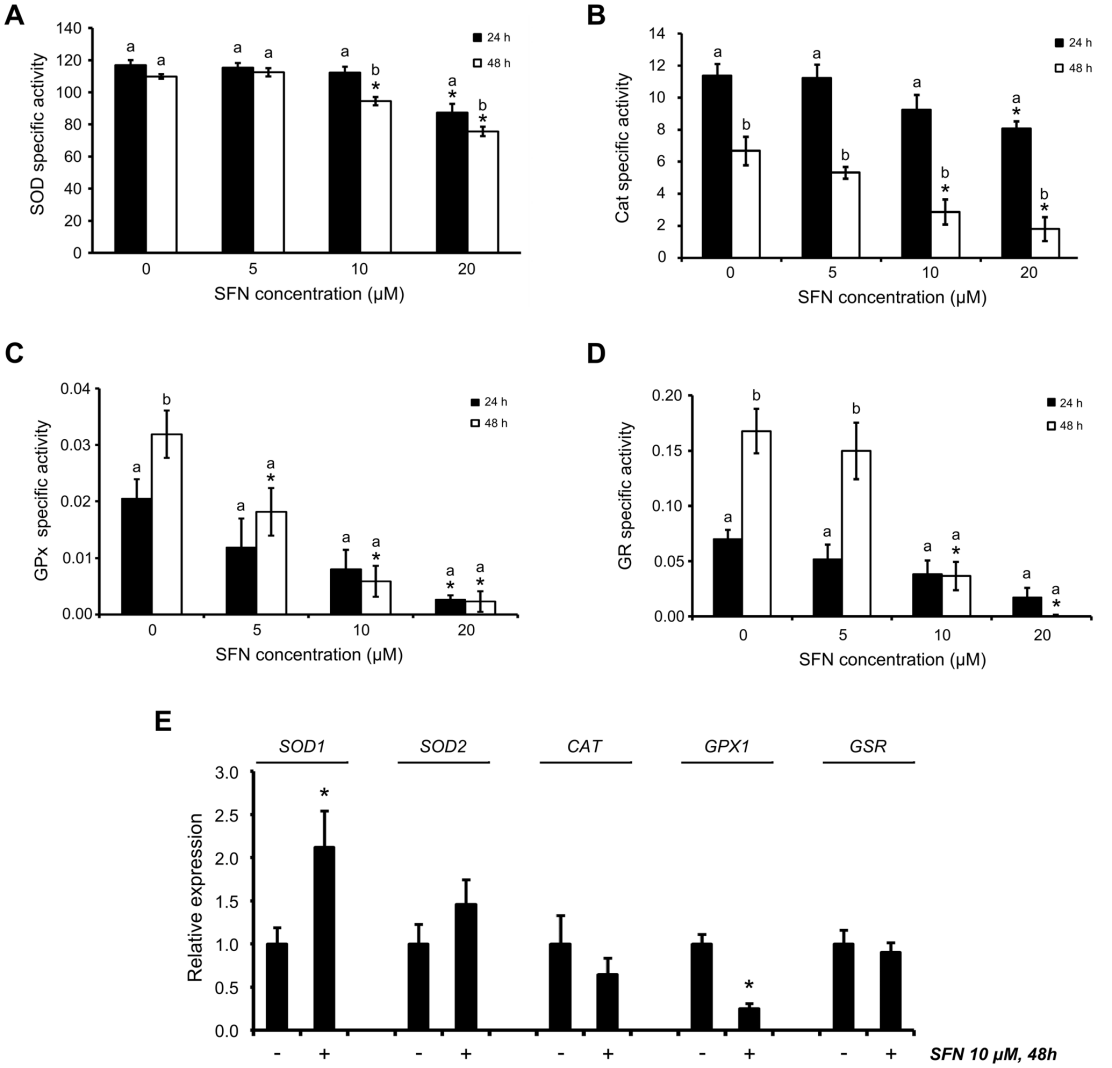
Effect of SFN on the activity and gene expression of selected oxidoreductases involved in ROS detoxification and GSH regeneration. (A–D) SOD, Cat, GPx and GR specific activities, respectively. Data shown are mean in U/mg total protein ± SEM (n = 3). *, significantly different between control and SFN-treated cells (*p*<0.05). a,b, significantly different between times (*p*<0.05). (E) Relative gene expression for cells exposed to 10 μM SFN for 48 h. Data shown are mean ± SEM (n = 2). *, significantly different between control and SFN-treated cells (*p*<0.05).

SFN induced a decrease in the activity of ROS-scavenging enzymes and enzymes involved in GSH regeneration (Fig. 5A–D). Increased ROS formation was associated with decreased SOD, GPx and GR enzyme activities, as given by correlation analysis (*p*<0.05; Table 2).

For the ROS-scavenging enzymes SOD, Cat and GPx, enzyme activity was significantly inhibited by higher SFN concentrations (Fig. 5A–C). SOD activity was affected by time only at higher SFN concentrations of 10 μM and above. Cat activity was affected by time independently of SFN treatment, since in control cells Cat activity decreased by 40% from 24 h to 48 h. Nevertheless, in cells exposed to 20 μM SFN, Cat activity decreased by 80% for the same period, thereby suggesting an additional effect caused by SFN treatment. In cells exposed to 10 μM SFN for 48 h, *SOD1* and *SOD2* gene expression was increased, whereas *CAT* expression was decreased (Fig. 5E). To some extent, this observation may explain the more pronounced effects of SFN in decreasing Cat activity compared to SOD activity. Upon SFN treatment, GPx gene expression and enzyme activity were both found significantly decreased and showed strong correlation with ROS accumulation in cells (*p*<0.01; Table 2). Moreover, apart from decreased GPx activity, SFN treatment resulted in decreased GR activity, suggesting that GSH regeneration was overall less effective in cells exposed to 10 μM or higher SFN concentrations. Moreover, for the lowest SFN concentration, i.e.5 μM, the decrease in enzyme activity was larger for GPx compared to GR, and this potentially stimulated the increase in intracellular GSH levels that was not found at higher SFN concentrations.

## Discussion

Compared to what is documented for other tumours, SFN cytotoxicity against osteosarcoma cells is still poorly studied and its effects on the oxidative state of osteosarcoma have remained uncharacterised. At 10 μM concentration or higher, SFN decreased cell viability, increased the%early apoptotic cells and increased caspase 3 activity. In previous work using identical conditions, a decrease in viability was already found at 5 μM SFN, as assessed by the 3-(4,5-dimethyl-2-thiazolyl)-2,5-diphenyl tetrazolium bromide (MTT) assay which assays mitochondrial activity [37].

In a study by Matsui and colleagues, SFN was shown to act as a sensitiser to TRAIL-induced apoptosis through DR5 receptor increased expression, in two p53 null osteosarcoma cell lines, including MG-63 [17]. Moreover, in hepatoma cells, Kim and co-workers showed that Cat overexpression almost completely blocked TRAIL-induced apoptosis both in p53 wild-type and mutant cells [22]. These observations served as basis for this study, in which independent apoptotic markers such as the presence of cell surface phosphatidylserine or caspase-3 activation revealed positive correlation with ROS accumulation in MG-63 cells. In this study, the%early and late apoptotic cells was found significantly increased with SFN doses, compared to control. Between 5 and 10 uM SFN,%of early apoptotic cells increased 2.5 fold for 24-h exposure and 2.8 fold for 48-h exposure. The increase in%apoptotic cells was nevertheless not linear with SFN dose for all SFN concentrations tested. Rather, a plateau seems to occur at higher doses, although SFN doses above 20 μM were not tested to confirm this. A concentration-dependent effect of SFN cannot be ruled out and further conclusions based on threshold assumptions in SFN studies must be regarded carefully. Tumour cells typically generate higher ROS levels compared to normal cells, e.g. [38], so this study investigated the association between ROS production and different endpoints in the presence of SFN. Pearson correlation test supports (Table 2) the hypothesis that the increase of apoptosis is correlated with the increase in ROS levels, which was steeper between 5 and 10 μM (Fig. 4).

Several studies have shown that lower SFN concentrations typically up to 5 μM SFN increase the intracellular GSH pool in many cell lines [39], [40], [41], [42]. However, additional reports have revealed that depending on cell line, higher SFN concentrations rapidly and markedly deplete the intracellular GSH levels [23], [43]. In this work, a dual response was observed for the intracellular GSH levels, characterised by an increase in GSH levels at 5 μM SFN exposure, followed by a significant decrease for the higher concentration tested. Hu and coleagues showed that SFN inhibits GR in A549 cell line and in cell-free systems, cell and proposed direct covalent binding to cysteine catalytic residues as the main inhibition mechanism [44]. Our results agree with the observations of Hu and colleagues for GR inhibition, however, in the study from Hu and colleagues SFN did not significantly decrease GPx expression or enzyme activity in A549 cells, unlike what was found in our study with MG-63 cells. The decreased GPx expression and activity in MG-63 cells exposed to SFN compared to A549 from the study of Hu and colleagues could point to different sensitivities of cell lines to oxidative stress regulation. The main enzymes responsible for peroxide detoxification are Cat and GPx. Under the conditions tested, these enzymes showed significantly lower activity at 10 uM SFN for 48 h, with GPx already decreased at 5 uM SFN for 48 h. These results are also reflected at the gene expression, with Cat expression decreased, and GPx1 expression significantly decreased. SOD activity was not so significantly affected by SFN in percentage and one possible reason for this is suggested by the gene expression results, since SOD genes were found more expressed in the experimental condition. Gene expression quantification for all conditions and both exposure times might provide more insight into the variation of corresponding gene expression.

Apart from direct reaction of SFN with GSH, the decreased GPx and GR activities, and consequent poor GSH regeneration, might also explain the lower GSH levels found at higher SFN concentrations.

From the%viable cells, ROS levels and SOD, Cat, GPx enzyme activities, it may be concluded that exposure to 10 μM SFN for 48 h produced significant pro-oxidant effects. Several genes are transcriptionally activated by Nrf2. In order to better understand the general antioxidant defences of MG-63 cells, the Nrf2-ARE response was analysed. TXNRD1 and NQO1, known to be positively regulated by Nrf2, were found expressed at higher levels upon exposure to SFN and TXNRD1 was significantly overexpressed; however, the two glutathione transferase genes studied which are also under the regulation of Nrf2 were not overexpressed. These results suggest that activation by Nrf2 was not complete under the given experimental conditions, and that the antioxidant response was limited.

Besides eliciting a dual response in GSH levels, exposure to increasing SFN concentrations resulted in a non-linear steep increase in intracellular ROS levels between non-exposed control and cells exposed to10 μM SFN. SFN is known to induce changes in the intracellular redox balance and depending on its concentration, exposure time or the exposed cell line, it may promote antioxidant or pro-oxidant response. *In vitro*, a predominantly antioxidant response has been reported at low SFN concentrations, e.g. up to 5 μM SFN for up to 24 h, or alternatively higher SFN concentrations for only few hours exposure [40], [42], [45], [46], [47]. On the other hand, and notwithstanding a possible antioxidant response, enhanced ROS accumulation has been previously documented in cells exposed to higher SFN concentrations, e.g. above 5 μM, or to long-lasting exposure periods, typically above the 24 h [26], [27], [42], [47], [48]. Moreover, it has been previously observed that SFN even at lower doses can be cytotoxic to multiple myeloma, suggesting that a subgroup of cancer cells may be extremely susceptible to SFN cytotoxic effects [49]. In the present study, the cytotoxic and pro-oxidant effects of SFN were found to be dependent on concentration and exposure period, although not following a linear correlation, and this deserves further investigation in *in vivo* experiments evaluating the role of SFN as coadjuvant in chemotherapy, prior to human trials.

**Figure 6:**
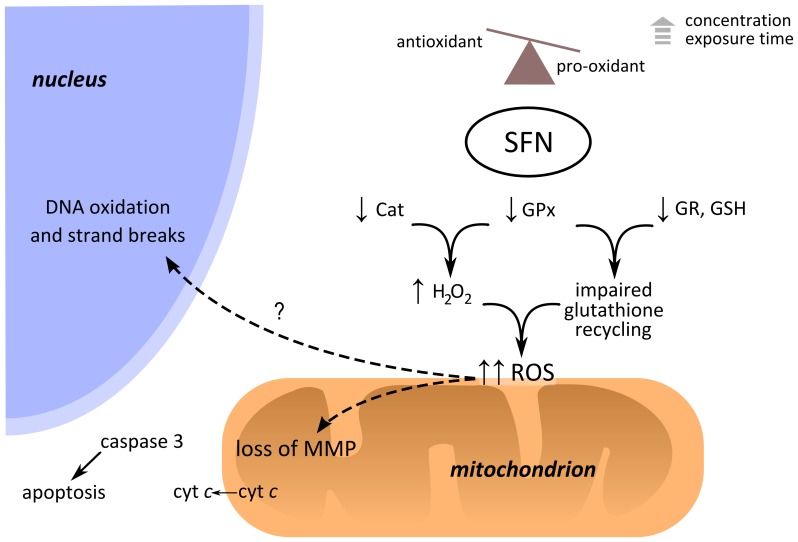
Hypothetical roles of SFN in ROS initiation and cytotoxicity in MG-63 cells. ROS accumulation and cell death after SFN treatment. ROS may accumulate as a consequence of combined decrease in GPx and Cat activities, together with overall decreased efficiency in glutathione recycling and GSH regeneration. ROS accumulation may additionally decrease the mitochondrial membrane potential, leading to apoptosis. MMP: mitochondrial membrane potential.

In the case of osteosarcoma, this study reveals different alterations leading to increased oxidative stress induced by SFN (summarised in Fig. 6).

As hypothesised in scheme for p53 null cells (Fig. 6), the ROS levels induced by SFN may contribute to effects such as G2/M phase arrest, loss of mitochondrial membrane potential (MMP) and p53-independent apoptosis. It is well known that cancer cells have higher glycolytic fluxes compared to normal cells and mitochondrial dysfunction in cancer is often related to this preferential use for glycolysis. In this respect, mitochondrial dysfunction and defective oxidative phosphorylation often associate with cancer cell survival and proliferation. Although the interplay between p53 action and ROS generation is complex and yet to decipher in p53 dysfunctional cells [e.g. 50], it would be expected that p53-null tumour cells have decreased mitochondrial respiration and generate less ROS. However, it has been demonstrated that very often cancer cells (independently of the p53 status) produce higher basal levels ROS, compared to normal cells. Since cancer cells are subjected to high oxidative stress, they may be more adapted and cope with small increments in ROS levels. Nevertheless, a small increment in ROS levels may render cancer cells more prone to deleterious events than the same ROS increase in normal cells, as shown e.g. by the selective killing of cancer cells promoted by the ROS-inducing piperlongumine [51] but this hypothesis requires further confirmation for the action of SFN on MG-63 oxidative stress and cell death compared to normal cells.

As shown in the hypothetical model (Fig. 6), SFN-related deleterious effects may include e.g. DNA strand breaks, membrane damage, and apoptosis induction [37]. Noteworthy, doxorubicin, an anticancer drug commonly used in osteosarcoma therapy, has been previously shown to induce intracellular ROS formation, which was found necessary for mitochondrial membrane depolarization, pro-caspase 3 activation, apoptosis and G2/M phase arrest in the p53-null SaOS-2 osteosarcoma cell line [52]. However, unlike doxorubicin which is cardiotoxic, SFN is still not reported to present cardiotoxicity at the doses presented in this study [53], [54].

In conclusion, from the toolbox of biomarkers tested, GPx expression and activity were found the most sensitive endpoints for prediction of oxidative stress in the studied model system. Despite the complex regulation and multiple interactions of SFN with different biomolecules, the reported anticancer mechanism is of potential interest to osteosarcoma therapy and deserves further investigation for this and other p53-null cancer cells that are chemoresistant.
